# Exploring the multifaceted potential of (R)-ketamine beyond antidepressant applications

**DOI:** 10.3389/fphar.2024.1337749

**Published:** 2024-04-11

**Authors:** Senbing Zhang, Yanzhu Pu, Jianning Liu, Lewen Li, Chibing An, Yumin Wu, Wenjie Zhang, Wenxia Zhang, Song Qu, Wenjun Yan

**Affiliations:** ^1^ The First Clinical Medical College, Gansu University of Chinese Medicine, Lanzhou, China; ^2^ Department of Anesthesiology, Xianning Central Hospital, The First Affiliated Hospital of Hubei University of Science and Technology, Xianning, Hubei, China; ^3^ Department of Anesthesiology, Gansu Provincial Hospital, Lanzhou, Gansu, China

**Keywords:** (R)-ketamine, antidepressant efficacy, treatment-resistant depression (TRD), side effects, ischemic stroke, cognitive disorders

## Abstract

(*R*, *S*)- and (*S*)-ketamine have made significant progress in the treatment of treatment-resistant depression (TRD) and have become a research focus in recent years. However, they both have risks of psychomimetic effects, dissociative effects, and abuse liability, which limit their clinical use. Recent preclinical and clinical studies have shown that (*R*)-ketamine has a more efficient and lasting antidepressant effect with fewer side effects compared to (*R*, *S*)- and (*S*)-ketamine. However, a recent small-sample randomized controlled trial found that although (*R*)-ketamine has a lower incidence of adverse reactions in adult TRD treatment, its antidepressant efficacy is not superior to the placebo group, indicating its antidepressant advantage still needs further verification and clarification. Moreover, an increasing body of research suggests that (*R*)-ketamine might also have significant applications in the prevention and treatment of medical fields or diseases such as cognitive disorders, perioperative anesthesia, ischemic stroke, Parkinson’s disease, multiple sclerosis, osteoporosis, substance use disorders, inflammatory diseases, COVID-19, and organophosphate poisoning. This article briefly reviews the mechanism of action and research on antidepressants related to (*R*)-ketamine, fully revealing its application potential and development prospects, and providing some references and assistance for subsequent expanded research.

## 1 Introduction

In 1964, the short-acting anesthetic, analgesic, sympathomimetic, and dissociative effects of (*R*, *S*)-ketamine (ketamine) were first discovered in human trials (Domino et al., 1965). This was followed by immeasurable contributions in numerous clinical practices and scientific studies (Conahan, 1975; Domino, 2010; Li and Vlisides, 2016; Tyler et al., 2017). With its combined analgesic and sedative effects, it had been widely used in surgical anaesthesia and adjuvant analgesic therapy (Gao et al., 2016; Mion, 2017; Barrett et al., 2020), and especially exerted unique advantages in the relief of various acute and chronic pains (Schwenk et al., 2018; Barrett et al., 2020; Yang et al., 2020). However, due to its psychedelic and psychomimetic effects, induction of postoperative nightmares, and abuse liability, it experienced a period of decline in clinical use (Mion, 2017; Wei et al., 2020). In 2000, Berman et al. (Berman et al., 2000) first showed in seven patients with major depressive disorder (MDD) that (*R*, *S*)-ketamine had rapid and sustainable antidepressant effects. This discovery was considered a major breakthrough in over 50 years of depression research and reignited the interest of the medical community in (*R*, *S*)-ketamine and its two enantiomers [(*S*)-ketamine (esketamine) and (*R*)-ketamine (arketamine)]. In 2019, the (*S*)-ketamine nasal spray (Spravato), one of the enantiomers of (*R*, *S*)-ketamine, was licensed in the US and Europe to treat treatment-resistant depression (TRD). However, due to the need for further validation of its efficacy, safety, and abuse risk, its widespread use is limited, primarily distributed by a controlled system with clear risk assessment and mitigation strategies (Turner, 2019; Jelen et al., 2021).

Given the issues of (*R*, *S*)- and (*S*)-ketamine in the therapy of depression, researchers began to focus on the other enantiomer of ketamine, (*R*)-ketamine. They found that in contrast to (*R*, *S*)- and (*S*)-ketamine, (*R*)-ketamine not only did not induce psychomimetic-like symptoms but also produced a state of relaxation and a feeling of wellbeing in healthy subjects (Vollenweider et al., 1997), while at the same time having potent, long-lasting antidepressant effects with fewer side effects (Hashimoto, 2020; Wei et al., 2021b; Scotton et al., 2022). Chang et al. (Chang et al., 2019) showed in their comparative study that in the mouse chronic social defeat stress (CSDS) model, the antidepressant potency of ketamine and its two enantiomers was in the order of (*R*)-ketamine > (*R*, *S*)-ketamine > (*S*)-ketamine. What’s more, in other animal models of depression, researchers similarly confirmed that the antidepressant effect of (*R*)-ketamine was observed to be more effective and prolonged than that of (*R*, *S*)-ketamine and its other metabolites, such as (*S*)-ketamine and (*2R*,*6R*)-hydroxynorketamine (HNK) (Zhang et al., 2014; Yang et al., 2015; Fukumoto et al., 2017; Shirayama and Hashimoto, 2018; Yang et al., 2018; Johnston et al., 2023). However, a recent small-scale clinical trial indicated that the effects of (*R*)-ketamine on TRD patients were not superior to those of the placebo group (Leal et al., 2023). This contrasting result has drawn widespread attention and interest in the industry, and also raised questions about the actual antidepressant efficacy of (*R*)-ketamine.

Moreover (Wang et al., 2022b), previously reviewed the potential preventive and therapeutic effects of (*R*)-ketamine in neurological conditions like Alzheimer’s disease (AD), other dementias, Parkinson’s disease (PD), multiple sclerosis (MS), and ischemic stroke. However, we have found through further reading of the literature on (*R*)-ketamine that the extended research on (*R*)-ketamine is not limited to depression and neurological diseases. It also shows potential applications in perioperative anesthesia, osteoporosis, substance use disorders, inflammatory diseases, COVID-19, and organophosphate poisoning, and most of the mechanisms of action are inextricably linked to their antidepressant mechanisms. In this review, we briefly overview the current status and mechanisms of action of (*R*)-ketamine in antidepressant research. We also specifically review preclinical and clinical studies comparing the efficacy of (*R*)-ketamine with other antidepressants, metabolites, and placebos. This aims to resolve controversies over the antidepressant efficacy of (*R*)-ketamine and to guide further research in TRD and MDD patients. Additionally, we have compiled the latest research on (*R*)-ketamine in non-depressive treatments to guide its other potential applications and future research directions.

## 2 Research on the antidepressant effects of (*R*)-ketamine

### 2.1 Preclinical studies

Although the affinity of (*R*)-ketamine for the N-methyl-D-aspartate receptor (NMDAR) (inhibition constant Ki = 1.40 μmol/L) is 4-fold lower than that of (*S*)-ketamine (Ki = 0.30 μmol/L), in rodents, (*R*)-ketamine exhibits a stronger and more long-lasting antidepressant effect than (*S*)-ketamine, with fewer psychomotor side effects and a lower risk of abuse (Zhang et al., 2014; Yang et al., 2017a; Fukumoto et al., 2017; Chang et al., 2019). Additionally, (*R*)-ketamine shows a stronger and longer-lasting antidepressant effect than (*R*, *S*)-ketamine and the NMDAR antagonist Lanicemine (Ebert et al., 1997; Yang et al., 2015; Zanos et al., 2016; Yang et al., 2018). Moreover, this effect does not lead to a significant rise in the medial prefrontal cortex’s (mPFC) dopamine (DA) release (Ago et al., 2019; Zhou et al., 2021) reported that the bilateral lateral habenular nucleus (LHb) administration of 25 μg/μL (*R*)-ketamine and (*2R*, *6R*)-HNK in rats (1 µL/side) did not significantly produce an antidepressant-like effect at 1 or 24 h. Thus, we can speculate that (*R*)-ketamine’s antidepressant effects might not be related to mechanisms such as NMDAR blockade, LHb neuronal firing, or DA receptor activation.

Further research indicated that α-amino-3-hydroxy-5-methyl-4-isoxazolepropionic acid receptor (AMPAR, one of the ionotropic glutamate receptors) antagonist NBQX, the transforming growth factor-β1 (TGF-β1) inhibitors RepSox and SB431542, the colony-stimulating factor 1 receptor (CSF1R) inhibitor PLX3397, and the γ-aminobutyric acid type A receptor (GABA_A_R) agonist muscimol can all inhibit the antidepressant effects of (*R*)-ketamine in animal models of depression. This suggests that the activation of TGF-β1 signaling pathway, CSF1R, AMPAR, and the inhibition of GABA_A_R are crucial for the rapid and long-lasting antidepressant effects of (*R*)-ketamine (Yang et al., 2015; Yang et al., 2018; Zhang et al., 2020; Rafało-Ulińska et al., 2022; Tang et al., 2023). Additionally, when compared to (*S*)-ketamine, the sustained antidepressant effects of (*R*)-ketamine may be primarily mediated through the nuclear receptor binding protein 1 (NRBP1), which is found in the microglial cells of the mPFC in adult mice. This is because (*R*)-ketamine was reported to activate ERK in primary microglial cells, thereby increasing the expression of NRBP1, brain-derived neurotrophic factor (BDNF), and the phosphorylated cAMP response element binding protein (p-CREB)/CREB, leading to its long-lasting antidepressant effects (Yao et al., 2022).

On the other hand, (*R*)-ketamine, through the BDNF-tropomyosin receptor kinase B (TrkB) signaling pathway, helps to restore the decreased BDNF levels in the prefrontal cortex (PFC), hippocampal CA3, and dentate gyrus (DG) regions of rodents (Yang et al., 2015; Yang et al., 2018; Fujita et al., 2020; Tan et al., 2020; Fujita et al., 2021; Lin et al., 2021; Qu et al., 2021). Simultaneously, (*R*)-ketamine can elevate the release of 5-hydroxytryptamine (5-HT) in the mPFC (Ago et al., 2019), and significantly inhibit the overexpression of the nuclear factor of activated T-cells 4 (NFATc4) signaling gene in the PFC (Ma et al., 2022c). These findings indicate that activating the BDNF-TrkB, NFATc4 signaling pathways, and endogenous 5-HT receptors might be key mechanisms for the antidepressant effects of (*R*)-ketamine.

In addition, activation of mammalian target of rapamycin (mTOR) and extracellular signal-regulated kinase (ERK) has been suggested as a hypothetical molecular mechanism for the antidepressant effects of ketamine (Tang et al., 2015; Zanos et al., 2018; Johnston et al., 2020). However, in CSDS model, (*R*)-ketamine significantly reversed the reduction of ERK and upstream effector mitogen-activated protein kinase/ERK kinase phosphorylation in the PFC and hippocampal DG of susceptible mice after CSDS, but not mTOR and its downstream effector ribosomal protein S6 kinase phosphorylation in the PFC. Meanwhile, mTOR inhibitors (rapamycin or AZD8055) also failed to block the antidepressant effects of (*R*)-ketamine (Yang et al., 2018).

However, in a chronic unpredictable mild stress (CUMS) model, (*R*)-ketamine was found to increase protein expression levels of phosphorylated mTOR (pmTOR) in the mouse PFC, reversing the CUMS-induced decrease in the pmTOR/mTOR ratio, but had no effect on ERK phosphorylation levels (Rafało-Ulińska and Pałucha-Poniewiera, 2022). These two diametrically opposed results may stem from different models of depression, study methods, dosing regimens, and timing of tissue collection, all of which may have a differential impact on the test results, and more in-depth studies will be needed in the future to arrive at more definitive answers.

Considering that the pathophysiology of MDD involves microRNAs (miRNAs) as a critical regulatory component of synaptic plasticity, this suggests that directly targeting miRNAs might be a potential therapeutic strategy for MDD (Zhou L. et al., 2021; Ma et al., 2022b) found that chronic restraint stress (CRS)-exposed mice could have their body weight loss, forced swimming test immobility duration, and sucrose preference greatly improved by giving (*R*)-ketamine (10 mg/kg) as a pretreatment 1 day prior to CRS. It also markedly attenuated the expression of miR-132-5p and its associated regulatory genes [BDNF, Methyl CpG binding protein 2 (MeCP2), Tgfb1, TGF-β receptor II (Tgfbr2)] in the PFC of mice given CRS. Additionally, the onset and progression of depression are closely associated with the upregulation of endoplasmic reticulum stress-related genes (Nevell et al., 2014). Therefore (Jóźwiak-Bębenista et al., 2022), compared the effects of two enantiomers of ketamine on the expression of endoplasmic reticulum stress-responsive genes in a human astrocyte cell line. The study found that (*R*)-ketamine has a relatively mild effect on the expression of genes in the unfolded protein response (UPR) pathway (Jóźwiak-Bębenista et al., 2022). Furthermore, it can also increase the expression of CREB3L1 in old astrocyte specifically induced substance (OASIS) encoded in astrocytes and CREB3/LUMAN mRNA in astrocytes, suggesting that the antidepressant effects of (*R*)-ketamine may be related to members of the OASIS family (Jóźwiak-Bębenista et al., 2022).

In addition, (*R*)-ketamine has anti-inflammatory effects (Zhang et al., 2021a; Zhang et al., 2021b), and significantly ameliorates increased spleen weight in CSDS-susceptible mice (Zhang et al., 2020; Wei et al., 2021a), resulting in amelioration of increased expression of the natural killer cell-activated receptor (NKG2D) in the spleen (Zhang et al., 2021). It can also partially repairs alterations in the gut microbiota that may be related to the onset and progression of certain diseases (Yang et al., 2017b; Qu et al., 2017; Wang et al., 2021; Wang et al., 2022a). This implies that the antidepressant mechanism of (*R*)-ketamine might be related to the brain-spleen axis and the microbiota-gut-brain axis (Getachew et al., 2018; Huang et al., 2019; Wilkowska et al., 2021; Wei et al., 2022).

Although the indirect metabolite of (*R*)-ketamine, (*2R*, *6R*)-HNK, has shown rapid and/or long-lasting antidepressant effects in various animal models of depression (Zanos et al., 2016; Chou et al., 2018; Pham et al., 2018; Zanos et al., 2019a; Zanos et al., 2019b; Fukumoto et al., 2019; Highland et al., 2019). Interestingly, some researchers did not observe the sustained antidepressant effects of (*2R*, *6R*)-HNK similar to (*R*)-ketamine (Yang et al., 2017a; Zhang et al., 2018a; Zhang et al., 2018c; Shirayama and Hashimoto, 2018; Yamaguchi et al., 2018; Xiong et al., 2019). Instead, they believe that (*R*)-ketamine produces its own antidepressant effects rather than being a result of (*2R*, *6R*)-HNK (Shirayama and Hashimoto, 2017; Yamaguchi et al., 2018). A study pointed out that (*2R, 6R*)-HNK is an inert molecule that differs from ketamine pharmacologically and does not bind to specific high-affinity sites in the brain (Bonaventura et al., 2022). Thus, it might produce antidepressant effects indirectly by interacting with physical or chemical processes or by weakly interacting with many different molecular targets in various biological systems (Bonaventura et al., 2022). Therefore, the precise antidepressant effects, mechanisms, and specific functions of (*2R*, *6R*)-HNK in the antidepressant effects of (*R*)-ketamine still require further research for confirmation.

**FIGURE 1:**
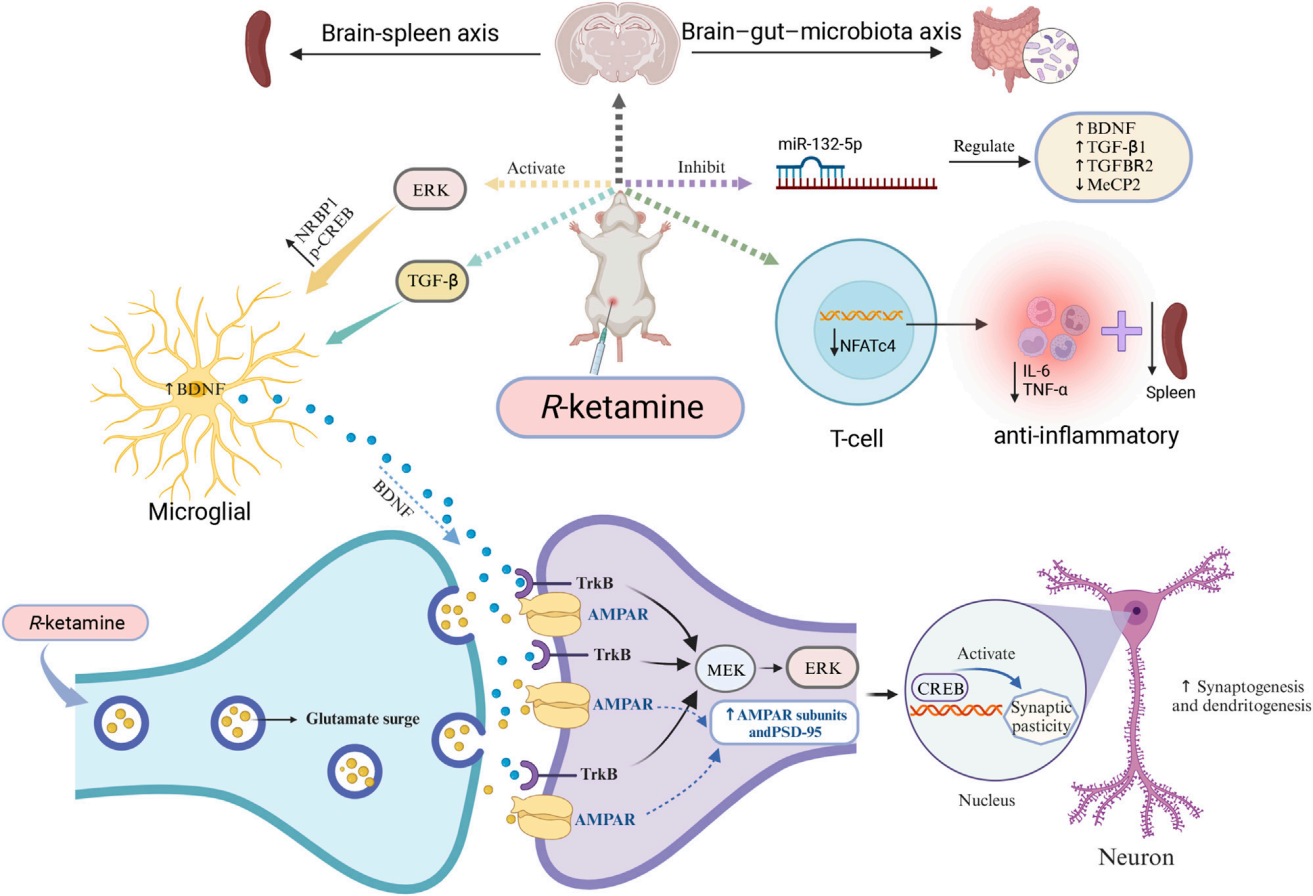
Mechanisms of (*R*)-ketamine antidepressant (partial) (*R*)-ketamine can activate ERK, thus increasing the expression of NRBP1, BDNF, and p-CREB in primary microglial cells. At the same time, it promotes the release of glutamate into the synaptic cleft, enhances AMPAR flux, activates cellular signaling, and increases the synaptic protein translation of AMPAR subunits and PSD-95, thereby promoting the formation of synapses and dendrites. Additionally, (*R*)-ketamine can regulate TGF-β signaling in microglial cells, increase the release of BDNF, and subsequently promote binding with the TrkB receptor. Through the MEK-ERK-CREB signaling pathway, it can enhance synaptogenesis and dendritogenesis, thus exhibiting its antidepressant effects. Furthermore, the antidepressant mechanism of (*R*)-ketamine may also involve the microbiome-gut-brain axis, the brain-spleen axis pathway, and the regulation of miR-132-5p and NFTc4. Abbreviations: AMPAR: α-amino-3-hydroxy-5-methyl-4-isoxazolepropionic acid receptor; BDNF: brain-derived neurotrophic factor; CREB: cyclic adenosine monophosphate response element-binding protein; ERK: extracellular signal-related kinase; MEK: mitogen-activated protein kinase; MeCP2: Methyl CpG binding protein 2. NFATc4: nuclear factor of activated T cells 4; PSD-95: postsynaptic density protein 95; TGF-β: transforming growth factor β; TrkB: tropomyosin receptor kinase B.

Although researchers have studied the antidepressant effects of (*R*)-ketamine from multiple perspectives, its specific mechanisms of action and exact target sites remain unclear. Clearly, these preclinical research findings provide guidance for subsequent studies and also offer theoretical support for clinical treatments and new drug development. (Figure 1 briefly illustrates some of the antidepressant mechanisms of (*R*)-ketamine).

### 2.2 Clinical research

Currently, all drug formulations of (*R*)-ketamine have not been approved for the market, but researchers continue to explore its antidepressant efficacy and side effects in clinical settings. In 2020, (Leal et al., 2021), initially documented a 40-min single intravenous infusion of (*R*)-ketamine (0.5 mg/kg) in 7 TRD subjects. By comparing the Montgomery-Åsberg Depression Rating Scale (MADRS) scores before and after the infusion, they observed that (*R*)-ketamine produced a rapid and significant antidepressant effect in TRD participants. This effect began to appear 60 min after the infusion, and peaked at 240 min, and 43% of the participants still showed antidepressant effects on the 7th day (Leal et al., 2021). Regarding side effects, only a portion of the participants briefly experienced mild blurred vision and dizziness, with no occurrences of dissociation or hemodynamic changes, indicating good safety of (*R*)-ketamine (Leal et al., 2021). Although this study yielded surprising antidepressant efficacy at the time, certain limitations in terms of the level of evidence for open-label design need to be noted. Therefore, the team redesigned a randomized, double-blind, crossover, pilot trial with a total sample size of 10 cases at 1-week intervals and treated all TRD patients with 0.5 mg/kg (*R*)-ketamine and saline intravenously (Leal et al., 2023). The results showed an improvement in depressive symptoms over time (2 weeks of observational analysis) and a lower incidence of adverse events in all TRD patients, but there was no significant difference between the (*R*)-ketamine group and the saline control group (Leal et al., 2023). This unexpected finding has once again raised questions about the actual antidepressant efficacy of (*R*)-ketamine. Compared to a previous open-label trial (Leal et al., 2021), subjects in this study had a longer duration of depression and more psychiatric co-morbidities such as obsessive-compulsive disorder, social anxiety disorder and generalized anxiety disorder (Leal et al., 2023). Based on the experience of previous ketamine antidepressant studies, some patients need to receive two or more doses of the medication in order to produce a response (Phillips et al., 2019; Phillips et al., 2020). Thus, a single (*R*)-ketamine administration may not achieve a level of depression treatment that would normally require a cumulative effect of the drug. In addition, a crossover design trial may not be optimal given the current uncertainty about the ideal therapeutic dose and frequency of administration of (*R*)-ketamine. Considering that the assessment of depression efficacy is largely dependent on MADRS scores, it is difficult to achieve significant between-group differences with a small sample size of 10 patients. Thus, although this small pilot study by LEAL et al. (Leal et al., 2023) failed to demonstrate that a single intravenous infusion of (*R*)-ketamine was superior to placebo in improving depression, the possibility that (R)-ketamine has antidepressant effects in humans cannot be completely discounted (*R*)-ketamine may provide positive clinical benefits, at least in terms of medication safety and adverse effects. Recent studies on (*R*)-ketamine in the treatment of bi-directional depression have again demonstrated its favorable antidepressant effects (Bandeira et al., 2023). In the study, six subjects with type I and type II bipolar disorders were treated with (*R*)-ketamine intravenously on two separate occasions (1 week apart) at doses of 0.5 mg/kg and 1 mg/kg, respectively). Before and after treatment, the subjects’ mean total MADRS scores were reduced by more than 50% and little dissociative and manic symptoms were observed at both doses, demonstrating the feasibility and safety of (*R*)-ketamine for its rapid antidepressant effect in the treatment of bipolar depression (Bandeira et al., 2023). This further emphasizes that (*R*)-ketamine, as a novel antidepressant with good development potential, may in the future require larger sample sizes, more flexibility in dosage and frequency of administration, as well as more study design options (such as parallel subgroup design, etc.) to gain insights into the actual antidepressant efficacy of (*R*)-ketamine in clinical practice.

In 2018, China registered and approved a large randomized controlled trial that will compare the safety and effectiveness of (*R*)-ketamine with (*S*)-ketamine and (*R*, *S*)-ketamine in TRD treatment (ChiCTR1800015879) (Chinese Clinical Trial Registry, 2019). The following year, China’s Hengrui Medicine Co., Ltd. received approval from the National Medical Products Administration and began clinical trials to treat refractory depression with hydrochloride (*R*)-ketamine nasal spray (Netease, 2019). On 19 February 2021, the American company, Perception Neuroscience, released its Phase I clinical research data on (*R*)-ketamine, showing that the dose of (*R*)-ketamine (PCN-101) required to produce similar perceptual changes is much higher than that of (*S*)-ketamine, and the total dose below 150 mg is safe and well-tolerated (Cision PR Newswire, 2021). At the same time, the company subsequently initiated a Phase II confirmatory trial for TDR patients, which will be a key study to evaluate the therapeutic effects and related side effects of (*R*)-ketamine (Cision PR Newswire, 2021).

Moreover, regarding the controversy in preclinical studies about the antidepressant efficacy of (*R*)-ketamine’s direct metabolite (*2R*, *6R*)-HNK, Grunebaum et al. (Grunebaum et al., 2019) found that 24 h after administering ketamine (0.5 mg/kg) intravenously to MDD patients with significant suicidal ideation, the patients had higher plasma concentrations of (*2R*, *6R*)-HNK, but this was not significantly related to clinical improvement in depression. The result suggests that we should be more cautious when evaluating the antidepressant effects of (*2R*, *6R*)-HNK (Grunebaum et al., 2019). Currently, a Phase I clinical trial for (*2R*, *6R*)-HNK is being conducted, which will provide an objective assessment of the actual antidepressant effects of (*2R*, *6R*)-HNK (ClinicalTrials, 2021).

Taken together, although (*R*)-ketamine has shown its distinct advantages in various animal models of depression, some clinical studies still have doubts about its actual antidepressant effects. To determine the specific antidepressant effects of (*R*)-ketamine and its metabolites, and to assess the safety, drug resistance, side effects, and abuse risks of medium-to long-term, high-dose use, we still need to rely on large-sample, multi-center, blind clinical randomized controlled trials, and compare various regimens in terms of dosing, frequency, and timing.

## 3 Research on non-depressive conditions

### 3.1 Cognitive impairments

There is growing evidence that cognitive deficits, especially mild cognitive deficits, are significantly present in childhood and adolescence prior to psychotic episodes (Mollon and Reichenberg, 2018). Cognitive deficits in the offspring of maternal immune activation (MIA) model mice in adulthood can be prevented by repeated intermittent use of (*R*)-ketamine (10 mg/kg/day, twice a week for 4 weeks) during adolescent and juvenile stages (P28-P56) and may reduce the risk of conversion to psychosis in adulthood through activation of the BDNF-TrkB signaling pathway in the brain (Tan et al., 2022). However, given the multiple concerns regarding the use of ketamine and its metabolites in adolescents, especially in infants and young children, translating the results of this study into clinical practice may take longer. In addition, long-term social isolation has been shown to potentially lead to social cognitive deficits (Beutel et al., 2017), and maintaining an individual’s social cognitive functioning typically involves two brain regions, the insula and prefrontal cortex (Menon and Uddin, 2010; Bicks et al., 2015). The unique activation of the anterior insula cortex (aIC) induced by (*R*)-ketamine (20 mg/kg, i.p.) restores the aIC function, which promotes the formation of social memories and ameliorates social cognitive impairment deficits in socially isolated reared mice (Yokoyama et al., 2024).

On the other hand, ketamine and its enantiomers have been shown to produce different effects on memory and cognitive function in individuals at different doses (Gill et al., 2021; Zhornitsky et al., 2022). It was shown that at a higher subanesthetic dose (20 mg/kg), both ketamine and its two enantiomers significantly impaired recognition memory in mice in the novel object recognition (NOR) test. Whereas at a lower dose (10 mg/kg), (*S*)-ketamine still produced significant impairment of recognition memory, but not (*R*)-ketamine (Ide et al., 2019). Furthermore, both ketamine and (*S*)-ketamine at doses of 10–20 mg/kg induced cognitive deficits in NMDA receptor subunit GluN2D knockout (GluN2D-KO) mice, but the same dose of (*R*)-ketamine did not inhibit cognitive functioning in GluN2D-KO mice (Ide et al., 2019), suggesting that, in terms of decreasing the impact on individual cognitive effects on memory, (*R*)-ketamine appears to have an advantage over ketamine and (*S*)-ketamine. In the future, there is still a need to further substantiate and explore the quantitative effects of various ketamine compounds on cognitive effects in human clinical studies.

The NMDAR antagonist phencyclidine (PCP) can cause schizophrenia-like symptoms, including cognitive impairments, in both healthy individuals and rodents. However, these cognitive impairments can be alleviated by intermittent use of (*R*)-ketamine twice a week for 2 weeks at a dose of 10 mg/kg/day rather than (*S*)-ketamine (Tan et al., 2020). Further, using (*R*)-ketamine in prevention and treatment can alleviate systemic and neuroinflammation in mice induced by lipopolysaccharide (LPS) and has preventive and therapeutic effects on delirium and cognitive impairments (Zhang et al., 2021b). Additionally, metabotropic glutamate receptors 2 and 3 (mGluR_2/3_) are considered potential drug targets for treating various neurological diseases (Li et al., 2022). Its selective inhibitors have demonstrated significant antidepressant effects with reduced side effects in research in preclinical and clinical settings (Jiang et al., 2023). Recent research demonstrated that when the mGlu2/3 receptor antagonist LY341495 was combined with (*R*)-ketamine, it not only reduced the side effects of (*R*)-ketamine, but was also effective against depression symptoms induced by CUMS. Combined treatment reversed CUMS-induced PFC-dependent memory deficits and restored the diminished strength of longterm potentiation (LTP), exerting characteristic antidepressant and cognitive-enhancing effects (Pałucha-Poniewiera et al., 2023). In addition, (*R*)-ketamine (7.5, 15, and 30 mg/kg, i.p.) dose-dependently increased electroencephalogram (EEG) theta power at 23 h of wakefulness and rapid eye movement (REM) sleep, further supporting its potential use in cognitive impairment (Pothorszki et al., 2024).

In addition, among healthy volunteers, (*R*)-ketamine has milder impacts on psychopathology and neurocognition to *(S)*-ketamine, which might produce a “negative experience”. Its potential “protective effect” can partially offset the adverse effects of (*S*)-ketamine (Passie et al., 2021). Patients who suffer from depression often encounter intense psychological distress and may have a distorted perception that “time is slowing or dilating”, and this perception is positively correlated with the intensity of suicidal ideation (Cáceda et al., 2020). However, unlike (*S*)-ketamine, the antidepressant effects of (*R*)-ketamine are not based on the underestimation of time or behavioral disorders, but might help enhance cognitive abilities (Popik et al., 2022). Considering that geriatric depression is closely related to all-cause dementia, AD, and vascular dementia (Zhao et al., 2022), and that dementia patients often exhibit anxiety, depression, and other neuropsychiatric symptoms (NPS) significantly associated with their overall cognitive abilities (Sabates et al., 2023), it is hypothesized that (*R*)-ketamine may have a useful preventive or delaying effect on the progression of late-life depression to dementia in patients. Furthermore, it may also have beneficial effects on the emotional and cognitive aspects of various types of dementia patients.

Therefore, synthesizing the above studies we can easily find that (*R*)-ketamine has a weak inhibitory effect on cognition, may improve cognitive dysfunction caused by other causes (such as psychiatric disorders) to a certain extent (Hashimoto, 2023), and may play a positive role in preventing or delaying the disease progression of AD, and it is expected to be a potential medication for preventing and treating cognitive deficits in patients in the future.

### 3.2 Perioperative anesthesia

Perioperative anaesthesia is one of the most prominent areas of clinical application of (*R*, *S*)-ketamine and (*S*)-ketamine in addition to the treatment of depression (Barrett et al., 2020). However, the use of (*R*)-ketamine in this area deserves deeper reflection, exploration and research. Indeed, (*R*)-ketamine was used in clinical trials as early as the 1980s, however, unfortunately, researchers at that time did not observe that (R)-ketamine exhibited stronger effects than (*R*, *S*)- and (*S*)-ketamine in hypnosis and analgesia (White et al., 1980; White et al., 1985). More recent studies, by administering an intravenous infusion of ketamine to healthy subjects and subsequently examining plasma metabolite concentrations at various time points, found that the plasma metabolite (*R*)-ketamine did not have any effect on the pain-relieving or dissociative effects of the racemate ketamine (Olofsen et al., 2022). The researchers thus hypothesized that (*R*)-ketamine may lack efficacy in pain relief (Olofsen et al., 2022). Considering these limited studies, the actual analgesic and sedative efficacy of (*R*)-ketamine has not been evaluated and supported by strong evidence due to limitations in trial design, original drug class, pain detection methodology, and subject population, and thus further confirmation from subsequent studies is still needed. In addition, unlike (*R*, *S*)- and (*S*)-ketamine, which induce cardiovascular side effects such as tachycardia, elevated blood pressure, and increased cardiac output to varying degrees (Geisslinger et al., 1993; Canuso et al., 2018; Zanos et al., 2018; Kamp et al., 2021), (*R*)- Ketamine is essentially unaltered in hemodynamic parameters in healthy subjects and is more advantageous in myocardial protection (Geisslinger et al., 1993; Leal et al., 2021; Olofsen et al., 2022). In studies of ketamine-related psychedelic side effects, elevated plasma concentrations of (*R*)-ketamine in humans do not significantly alter measured effects such as anti-injury perception and psychedelic symptoms originally induced by (*S*)- and (*R*, *S*)-ketamine (Olofsen et al., 2022). Instead, this may be synergistic with the nitric oxide (NO) donor sodium nitroprusside in reducing (*R*, *S*)-ketamine-induced psychedelic symptoms and internal and external perceptual deficits (Jonkman et al., 2018).

In addition, (*R*)-ketamine has shown elimination of anxiety-related behaviors and social interaction disorders induced by MIA (de Oliveira et al., 2021), as well as the ability to rapidly improve depression-like behaviors in rodents (Yang et al., 2018; Yao et al., 2022). This suggests that (*R*)-ketamine may also have some potential for development and application in alleviating preoperative anxiety and postoperative *postpartum* depression. Given the lower affinity of (*R*)-ketamine for the cytochrome P450 enzyme system, its drug clearance is 50% lower than that of (*S*)-ketamine, resulting in a longer retention and duration of action in the body (Kamp et al., 2020). Once the positive clinical efficacy and indications regarding the positive effects of (*R*)-ketamine in anxiolytic, antipsychedelic, cardioprotective, neuroprotective, and PND prevention and amelioration have been further demonstrated, its prolonged *in vivo* retention properties will be more conducive to a lasting perioperative effect.

### 3.3 Ischemic stroke

Ischemic stroke, also known as a stroke, is a frequent acute cerebrovascular illness that has high rates of morbidity and mortality (Collaborators, 2020). Very effective therapeutic drugs and methods are still limited currently (Oh et al., 2022). Research has shown that depression and anxiety were found to increase the risk of stroke (Nakada et al., 2023). Conversely, a stroke could also trigger and exacerbate symptoms of depression and anxiety (Chen et al., 2023). Early identification and prevention of post-stroke depression (PSD) can significantly improve patients’ depressive symptoms, promote the recovery of physical and cognitive functions, and thereby enhance the prognosis of stroke and the 10-year survival rate (Robinson and Jorge, 2016; Sun et al., 2023). It was reported that, in middle cerebral artery occlusion (MCAO) and chronic CUMS model rats, a single local injection of ketamine in the dentate gyrus region could enhance synaptic plasticity by regulating NMDAR/calcium/calcium-calmodulin-dependent protein kinase II (CaMKII), thereby producing significant and long-lasting antidepressant effects (Abdoulaye et al., 2021). Furthermore, ketamine, administered through intraperitoneal injection 30 min after MCAO, demonstrated significant reductions in infarct volume, edema ratio, and neurological deficit, providing neuroprotection against ischemic brain injury (Shu et al., 2012). Given that both ketamine and (*S*)-ketamine have shown improvements and neuroprotective effects against ischemic stroke (Shu et al., 2012; Zhang et al., 2023), (*R*)-ketamine, compared to ketamine and (*S*)-ketamine, has a stronger, more lasting antidepressant effect with fewer side effects (Johnston et al., 2023). Thus (Xiong et al., 2020), further confirmed that (*R*)-ketamine is also significant in ischemic stroke through animal experiments. The study demonstrated that after administering mice with (*R*)-ketamine (10 mg/kg) 30 min before (or 1 h after) MCAO and 24 h post-MCAO, it was observed that (*R*)-ketamine not only significantly alleviated brain injuries and behavioral abnormalities caused by MCAO but also showed stronger neuroprotective effects than (*S*)-Ketamine (Xiong et al., 2020). This suggests that (*R*)-ketamine may develop into a novel medication for the prevention and treatment of ischemic stroke or PSD.

### 3.4 Multiple sclerosis

MS is an immune-mediated disease that leads to inflammatory demyelinating lesions in the central nervous system, including the spinal cord and brain (Wang et al., 2021). Affected by early inflammation and delayed neurodegenerative lesions, the disability rate is extremely high (Dobson and Giovannoni, 2019). In MS patients, MDD and its severe symptoms are very common and are closely related to a significant reduction in quality of life and an increased risk of suicide (Jones et al., 2021). Wang et al.'s study (Wang et al., 2021) showed that after continuously injecting (*R*)-ketamine (10 mg/kg/day) into MS model mice for 15 days, the weight loss of the mice was significantly alleviated. Furthermore, unlike the saline, (*R*)-ketamine improved the clinical experimental autoimmune encephalitis (EAE) score of mice, attenuated their pathology scores, microglia activation, and the integrity of the blood-brain barrier in the spinal cord. To sum up, these findings indicate that there is a preventive effect of (*R*)-ketamine. On the other hand, cuprizone (CPZ) is often used to establish a mouse model that mimics the demyelination in MS patients due to its demyelinating effect. A recent study found that after continuously injecting mice with (*R*)-ketamine (10 mg/kg/day) twice a week for 6 weeks, it could improve the CPZ-induced corpus callosum demyelination and microglial cell activation by activating TrkB, promote myelin regeneration after discontinuing CPZ, and partially restored the abnormal β-diversity of the gut microbiota in mice treated with CPZ (Wang et al., 2022a). Therefore, these results imply that (*R*)-ketamine may be a potential drug for the prevention and treatment of MS, and the gut-microbiota-microglial crosstalk may significantly influence the effects of (*R*)-ketamine in the CPZ-treated MS model mice.

### 3.5 Parkinson’s disease

PD is a chronic progressive neurodegenerative disorder that primarily damages the neurons in the substantia nigra (SNr) responsible for producing dopamine. Although most of the current drugs can alleviate the motor symptoms of patients, they cannot provide adequate neuroprotection or halt the disease’s progression(Kieburtz et al., 2018). While the primary clinical manifestation of PD is motor symptoms, non-motor symptoms like anxiety and depression are also the most common and significant psychiatric features of PD (Landau et al., 2016). Moreover, these two symptoms often accompany PD patients and may persist throughout the disease course (Zhang et al., 2023). In addition, cognitive deficits related to learning and memory abilities have been observed in more than 15% of PD patients (Svenningsson et al., 2012). Subanesthetic doses (5, 10, and 15 mg/kg) of ketamine (i.p., once weekly) have all been shown to reverse depressive-like behavior, short-term memory impairment, pleasure deficits, and to improve gait deficits in rats with PD model induced by bilateral lesions in SNc (Vecchia et al., 2018; Vecchia et al., 2021). Meanwhile, ketamine at 8 mg/kg also exerted neuroprotective effects through the activation of cellular autophagy in PD model mice, which increased the number of nigrostriatal dopaminergic neurons (Fan et al., 2017). However, the potential risk of drug abuse and possible psychiatric side effects of ketamine are issues that need to be focused on in its future clinical treatment of PD. Fujita et al.’s study (Fujita et al., 2020) observed whether the two enantiomers of ketamine had neuroprotective effects in PD mice by 1-methyl-4-phenyl-1, 2, 3, 6-tetrahydropyridine (MPTP)-induced PD mouse model. The results showed that repeated intranasal administration of both (*R*)- and (*S*)-ketamine could effectively reduce the decline of dopamine transporter (DAT) in the striatum of mice caused by MPTP, with (*R*)-ketamine showing a more pronounced effect than (*S*)-ketamine (Fujita et al., 2020). Further, by continuously administering (*R*)-ketamine intranasally, tyrosine hydroxylase (TH) in the striatum and SNr decreased significantly as a result of MPTP, while (*S*)-ketamine did not have this effect (Fujita et al., 2020). In addition, BDNF provides trophic support through the TrkB signaling pathway to increase dendritic and axonal branching and synaptogenesis. This signaling pathway plays an important role in the development of neurodegenerative diseases such as PD and AD and psychiatric disorders such as depression, and has become a key target for the development of related therapeutic drugs (Hashimoto, 2010; 2013; Mitre et al., 2017). It has been shown that (*R*)-ketamine normalizes the protein levels of BDNF and p-TrkB in the PFC and hippocampal CA3 and DG regions of mice modeled for depression, promotes synaptogenesis, and ameliorates the reduction of dendritic spine densities in the prelimbic (PrL), CA3 and DG of the mPFC (Yang et al., 2015). Also, (*R*)-ketamine significantly attenuated MPTP-induced reduction of DAT in mouse striatum, and these ameliorative effects could be blocked by pretreatment with a TrkB antagonist (ANA-12) (Fujita et al., 2020). It is speculated that (*R*)-ketamine may play a neuroprotective role in counteracting MPTP-induced neurotoxic effects in the PD brain by activating the BDNF-TrkB signaling pathway in the striatum and SNr (Fujita et al., 2020), suggesting that it may be a possible new drug in the future for the prevention or treatment of neurodegenerative diseases such as Parkinson’s disease.

### 3.6 Osteoporosis

Osteoporosis is characterized by reduced bone density, deterioration of microarchitecture, and susceptibility to fragility fractures (Lane et al., 2000). MDD is one of the important risk factors for osteoporosis, and bone mineral density (BMD) is lower in both adults and women when combined with depression. Therefore, assessing and treating depression in these high-risk patients is crucial for preventing osteoporosis (Yuan et al., 2021). It was reported that the osteoprotegerin/nuclear factor κB receptor activator/nuclear factor κB receptor activator ligand (OPG/RANK/RANKL) signaling pathway and the osteopontin (OPN) system had essential involvement in bone metabolic abnormalities caused by MDD. Moreover, the antidepressant effects of (*R*, *S*)-ketamine might be associated with the improvement of inflammatory bone markers (Kadriu et al., 2018). Further research found that instead of (*S*)-ketamine and (*2R*, *6R*)-HNK, (*R*)-ketamine dramatically reduced the elevated plasma levels of RANKL and ameliorated the reduction in the OPG/RANKL ratio in CSDS susceptible mice (Zhang et al., 2018b; Xiong et al., 2019). Additionally, it could also significantly increase the BMD of the femur in CSDS susceptible mice (Xiong et al., 2019). In conclusion, it could be inferred that the OPG/RANKL ratio in the blood of MDD patients might be a potential biomarker for assessing the antidepressant effects of (*R*)- and (*R*, *S*)-ketamine, which is crucial for diagnosis (Zhang et al., 2018b). Wan et al. (Wan et al., 2022) reported that administering (*R*)-ketamine (10 mg/kg/day, twice a week) continuously for 6 weeks could dramatically enhance the cortical bone density and overall density of ovariectomized (OVX) mice by modulating the anti-inflammatory effects of gut microbiota. Similarly, one dosage of 10 mg/kg of (*R*)-ketamine improved anhedonia-like behavior, as well as reduced femoral neck cortical and total BMD. Furthermore, one dose of (*R*)-ketamine altered gut microbiota composition, resulting in changes in thirteen metabolic pathways and six metabolites in CSDS susceptible mice. The findings suggest that by acting on the gut–microbiota–bone–brain axis, (*R*)-ketamine can ameliorate the anhedonia-like phenotype and decrease BMD in CSDS susceptible mice (Wan et al., 2023). As a result, (*R*)-ketamine might emerge as a promising drug for treating reduced bone density or osteoporosis in patients with depression in the future.

### 3.7 Inflammatory disease

LPS can induce a phenotype similar to depression, causing systemic inflammation and increased spleen weight, so it is often used to establish inflammation-related rodent models of depression (Zhang et al., 2020). However, (*R*, *S*)-ketamine can attenuate or even reverse the inflammatory response of BV2 microglial cells caused by LPS (Lu et al., 2020) and reduce the levels of high mobility group protein B1 (HMGB1) in plasma and vital organs such as the heart, liver, and kidney, thereby increasing the 7-day survival rate of rats with sepsis caused by cecal ligation and puncture (CLP) (Zhang et al., 2014; Zhang et al., 2021a) further reported that the combined use of (*R*)-ketamine at 15 mg/kg for prevention and treatment significantly improved the 14-day survival rate of mice after CLP. Additionally, it can also improve the reduction in rectal temperature caused by sepsis 12 h after CLP, the elevated levels of plasma inflammatory cytokines, and the increase in injury markers of vital organs such as the heart, lungs, kidneys, and liver. All these point to (*R*)-ketamine’s possible effectiveness in the prevention and treatment of sepsis. On the other hand, pretreatment with 10 mg/kg of (*R*)-ketamine (6 days before LPS injection) or combined with treatments 24 h before and 10 min after LPS injection can effectively prevent inflammation and significantly reduce spleen enlargement, central and systemic inflammation, and cognitive dysfunction in mice caused by LPS (Zhang et al., 2021b; Ma et al., 2022c). Furthermore, pretreatment with (*R*)-ketamine at 10 mg/kg 6 days before LPS administration can effectively correct the aberrant expression of two miRNAs (miR-149 and miR-7688-5p) and NFATc4 mRNA in the PFC of mice induced by LPS, and partially restore the effects of LPS on the mouse gut microbiota, thereby continuously preventing depressive-like behavior in inflammation model mice (Ma et al., 2022a; Yao et al., 2022). Additionally, depression might increase the risk of inflammatory bowel disease, but using antidepressants to treat depression can reduce this risk (Frolkis et al., 2019). Fujita et al. (Fujita et al., 2021) found that repeated injection of (*R*)-ketamine (10 mg/kg/day, for 14 or 7 days) had positive effects on the ulcerative colitis (UC) model induced by dextran sulfate sodium (DSS) through the activation of TrkB. Together, these findings indicate that (*R*)-ketamine has significant anti-inflammatory effects, especially when administered multiple times or for prevention and treatment. Thus, it holds promise as a new drug choice for treating inflammatory diseases such as sepsis and UC.

### 3.8 COVID-19

Increasing evidence suggests that the novel coronavirus infection may have adverse effects on the central nervous system (CNS), causing psychiatric and neurological symptoms in those infected (Chen et al., 2022). The endoplasmic reticulum chaperone protein σ-1 receptor is crucial for the replication of the novel coronavirus in host cells and is considered a potential therapeutic target for COVID-19 patients (Gordon et al., 2020a; Gordon et al., 2020b). Research has found that (*R*, *S*)-ketamine not only has sedative, analgesic, and antidepressant effects but also has a minimal impact on respiration. It can interact with σ receptors (including σ-1 and σ-2) (Robson et al., 2012) and is believed to have potential benefits for the treatment of COVID-19 patients (Ortoleva, 2020; Akinosoglou et al., 2021). Moreover, compared to two other ketamine compounds, (*R*)-ketamine has a more pronounced effect on the σ-1 receptor (Hashimoto, 2021). It has significant advantages in improving inflammatory diseases of the central nervous system (e.g., the spinal cord and brain) as well as lung inflammation (Zhang et al., 2021a; Wang et al., 2021), and can help COVID-19 patients feel more comfortable (Vollenweider et al., 1997). Therefore, (*R*)-ketamine, with its dual anti-inflammatory and antidepressant effects, may benefit such patients and holds promise as a new therapeutic candidate for the future treatment of COVID-19.

### 3.9 Substance use disorder

The consumption of alcohol and illicit drugs represents a growing and intricate worldwide public health issue. Even though current behavioral interventions and drug treatments have achieved some results, the outcomes for treating substance use disorders (SUDs) are still not ideal (Jones et al., 2018). A systematic review found that using (*R*, *S*)-ketamine to treat SUDs can reduce patients’ craving, motivation, and use of cocaine. It can also increase the success rate of withdrawal from alcohol and opioids and maintain a significant difference from the control group for up to 2 years after a single dose (Jones et al., 2018; Witkin et al., 2020) demonstrated that on its own, (*R*)-ketamine did not cause conditioned place preference (CPP). In contrast, it blocked morphine-induced CPP and reduced overall morphine withdrawal scores in rats following naloxone-precipitation. Furthermore, it was reported that (*R*)-ketamine (20 mg/kg) could significantly alleviate morphine withdrawal signs, with its efficacy comparable to that of the commonly used opioid withdrawal drug, Lofexidine (Witkin et al., 2020). Pretreatment with (*R*)-ketamine at 3 mg/kg in rats showed a significant inhibitory trend towards tolerance induced by ethanol (ETOH), and it did not affect ETOH’s dependence or its effects (Shafique et al., 2021). Therefore, it can be seen that (*R*)-ketamine not only does not induce or exacerbate substance dependence but, on the contrary, can play a potential therapeutic role in ethanol- and opioid-induced SUDs. Future clinical randomized controlled trials will further validate its effects in treating substance use disorders.

### 3.10 Organophosphate poisoning

The repeated use of various low-dose nerve agent substitutes and organophosphate agents [diisopropyl fluorophosphate (DFP)] can simulate the chronic depressive state of Gulf War Illness (GWI) in rats, mainly manifesting as emotional and cognitive impairments (Phillips and Deshpande, 2016). Using this model, researchers administered a single dose of (*R, S*)-ketamine (10 mg/kg) to rats 3 months after exposure to DFP. The results showed that rats displayed antidepressant effects in the forced swim test 1 h after treatment, and this effect remained significant 24 h posttreatment (Ribeiro et al., 2020). Meanwhile, the reduced expression of BDNF in the rat brain due to DFP exposure did not show significant improvement 1 h after (*R*, *S*)-ketamine treatment, but 24 h later, the level of BDNF expression dramatically rose (Ribeiro et al., 2020). Additionally, in another experiment by the team, they found that (*R*, *S*)-ketamine and its two enantiomers could significantly ameliorate the GWI characteristic behaviors of DFP rats (Zhu et al., 2020). Furthermore, compared to (*R*, *S*)- and (*S*)-ketamine, the ameliorative effects of (*R*)-ketamine were more powerful, prolonged, and associated with less severe CNS adverse effects. (Zhu et al., 2020). Acetylcholinesterase inhibitors in organophosphate pesticides can affect cholinergic signaling modulation, and this cholinergic signal also involves the regulation of glutamatergic transmission (Picciotto et al., 2012). At sub-anesthetic doses, (*R*, *S*)-ketamine can elevate extracellular glutamate levels and glutamate cycling in rats (Zanos and Gould, 2018). Considering that the NMDA receptor family includes seven subunits: GluN1, GluN2A-D, and GluN3A-B, of which GluN2D is only associated with cognitive impairments and motor sensitization, as well as persistent antidepressant effects induced by (*R*)-ketamine, it is essentially unrelated to the effects of (*R*, *S*)-ketamine and (*S*)-ketamine (Ide et al., 2017; Ide and Ikeda, 2018; Ide et al., 2019). Therefore, (*R*)-ketamine may demonstrate its multifunctional effects in GWI by modulating glutamatergic signal transmission and is expected to become a novel choice for treating organophosphate poisoning.

## 4 Side effects and abuse potential of (*R*)-ketamine

(*R*)-ketamine demonstrates greater advantages in its antidepressant effects compared to ketamine and (*S*)-ketamine (Zhang et al., 2014; Fukumoto et al., 2017; Chang et al., 2019). However, in recent years, researchers have remained concerned about its potential for abuse and common side effects, such as psychotomimetic and dissociative effects, that are shared by ketamine and (*S*)-ketamine (Chang et al., 2019). studied the effects of ketamine and its enantiomers on spontaneous movement and pre-pulse inhibition (PPI) in the mouse CSDS model, finding the strength of these effects was ranked as follow: (*S*)-ketamine > (*R*, *S*)-ketamine > (*R*)-ketamine. At the same time, (*R*)-ketamine does not induce anhedonia-like effects (Witkin et al., 2020), nor does it cause acute hypermobility effects or significant PPI deficits (Yang et al., 2015). When administered at a dose of 20 mg/kg, it not only does not induce CPP in mice or increase their scores, but it also alleviates the dissociative and psychomimetic effects caused by (*R*, *S*)- or (*S*)-ketamine (Yang et al., 2015; Chang et al., 2019). In addition, in drug discrimination tests in rats, when the aim was to induce cognitive deficits, (*R*)-ketamine showed a specific discriminative stimulus effect, making drug discrimination challenging. Compared to (*S*)- and (*R*, *S*)-ketamine, the cognitive deficits it induced were milder (Popik et al., 2020). Nonetheless, Zanos et al. (Zanos et al., 2019a) pointed out that some previous conclusions might not be accurate, as many studies did not use a full range of relevant subanesthetic doses for testing. Zanos and his colleagues found that for CD-1 mice, the minimum effective anesthetic dose of (*R*)-ketamine was 120 mg/kg, while the subanesthetic dose was 90 mg/kg (Zanos et al., 2019a). Simultaneously, when the dose exceeded 20 mg/kg, (*R*)-ketamine caused motor discoordination in mice; at 40 mg/kg, it induced acute hyperlocomotion in mice and produced evident CPP; and at 90 mg/kg, it interfered with PPI. In conclusion, these suggest that (*R*)-ketamine may still induce various side effects and carry a risk of abuse when used in higher-than-antidepressant dosages (Zanos et al., 2019a).

Given that the addictive potential of ketamine and its enantiomers in different individuals seems to be closely related to its pharmacological properties and its impact on individual psychology (Shim, 2022), Bonaventura et al. (Bonaventura et al., 2021) analyzed the pharmacological and behavioral characteristics of (*R*)-ketamine and (*S*)-ketamine, finding that the two differ in the physiological mechanisms of drug dependence. Compared to (*R*)-ketamine, (*S*)-ketamine had a greater affinity and potency for opioid receptors (Bonaventura et al., 2021). However, repeated injections of a subanesthetic dose of (*S*)-ketamine (20 mg/kg, IP) led to significant psychomotor sensitization and CPP, while the behavioral changes caused by the same dose of (*R*)-ketamine were milder or almost negligible (Bonaventura et al., 2021). Thus, the pharmacological effects of (*S*)-ketamine might be the primary factor in the human abuse liability of racemic ketamine, whereas (*R*)-ketamine may not influence it (Bonaventura et al., 2021).

Recently (Shim, 2022), expressed doubts about the conclusion mentioned above, as a previous study reported that 10–30 mg/kg of (*R*)-ketamine significantly enhanced spontaneous motor activity in mice, surpassing the effects of 30 mg/kg of (*S*)-ketamine (Fukumoto et al., 2017). However, in their study (Bonaventura et al., 2021), administered acute injections of (*R*)-ketamine at 5, 10, and 20 mg/kg to mice, observing increased motor activity only at the 20 mg/kg dose, and its effect was weaker than that of (*S*)-ketamine at the same dose. Therefore, subsequent studies should delve into factors such as procedures, measurement timing, and species differences to explain the discrepancies in the studies above (Shim, 2022). On the other hand, in the mouse behavioral sensitization model, a mere 3-day observation period might be insufficient to conclusively determine whether low-dose repeated injections of (*R*)-ketamine lead to psychomotor sensitization. Thus, a longer treatment duration and lower doses should be used to accurately assess the effects of the two enantiomers on mice (Shim, 2022).

In summary, most current preclinical studies and some clinical research indicate that compared to (*R*, *S*)- and (*S*)-ketamine, (*R*)-ketamine exhibits milder effects in terms of psychosis-like symptoms, dissociative side effects, and abuse potential, with a higher safety profile (Geisslinger et al., 1993; Hashimoto, 2020; Wei et al., 2021b; Olofsen et al., 2022). However, when considering the abuse potential and psychomotor sensitization, the conclusions of existing research remain contentious. For this reason, future studies should involve large-sample, randomized, double-blind, placebo-controlled trials. These trials should encompass subjective evaluations of drug craving or preference, observations of behavioral sensitization from repeated dosing, and long-term follow-ups of patients with TRD and potential co-morbid SUDs to assess misuse; addiction; and psychomotor sensitization.

## 5 Conclusion

Due to the significant effects of ketamine in treating TRD, researchers have intensively studied its antidepressant molecular mechanisms over the past two decades, especially its two enantiomers, metabolites, functional characteristics, and selective targets. In spite of the fact that the etiology and mechanisms of MDD are not fully understood, the efficacy of antidepressants remains variable due to the complexity of the disease and individual differences in symptoms and gene expression profiles in patients with MDD (Xia et al., 2022). Additionally, although ketamine’s and its enantiomers’ benefits for treating MDD are widely recognized, the specific molecular mechanisms of its anti-depressant action remain unclear (Heifets et al., 2021). Furthermore, existing studies show that (*R*)-ketamine has significant advantages over other drugs in terms of antidepressant efficacy and side effects, but knowledge of its antidepressant mechanisms and targets of intervention remains limited. In the future, we need to integrate chemistry, biology, and genetic technology to further explore these mechanisms.

**FIGURE 2:**
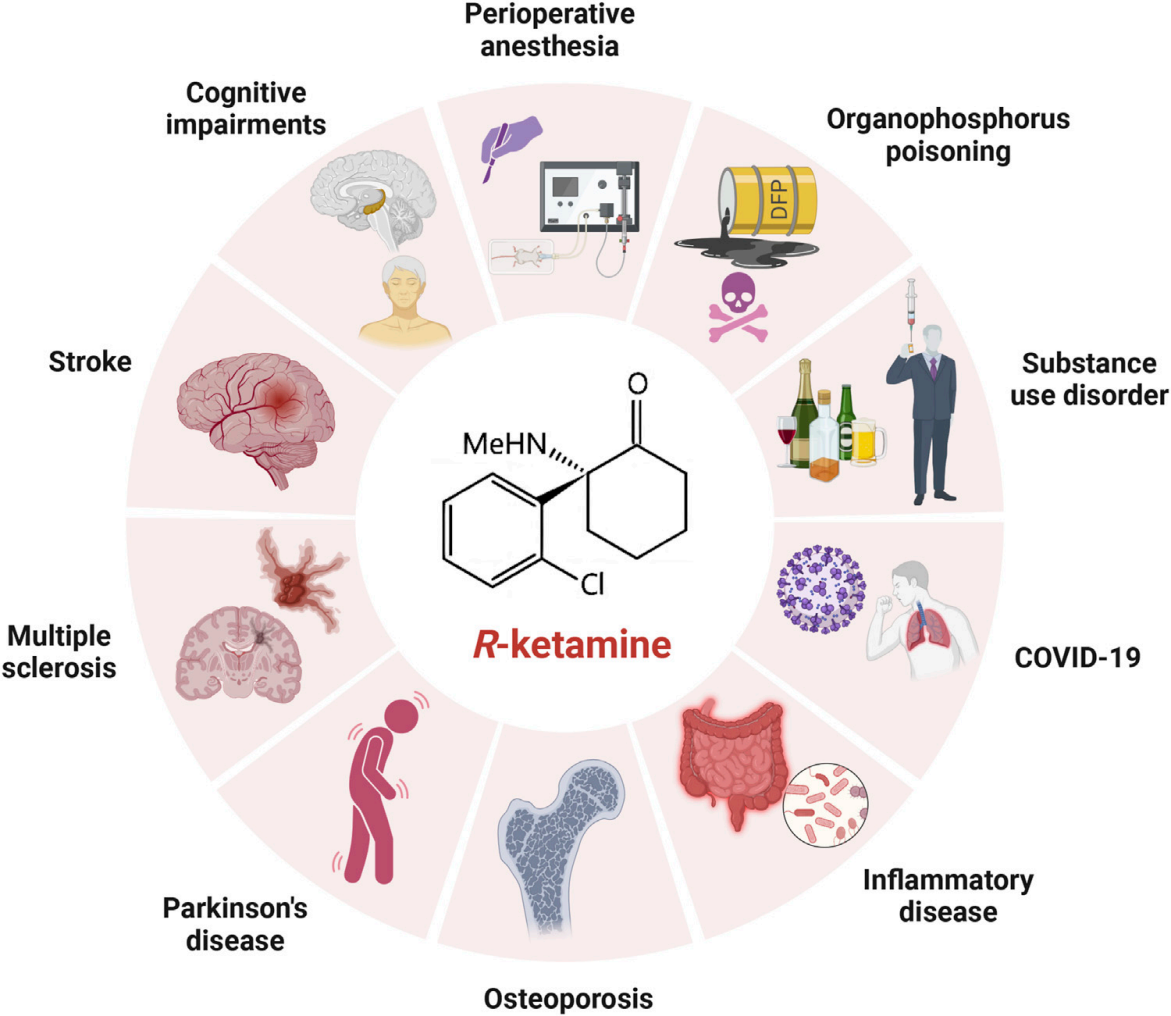
Applications of (*R*)-ketamine in treatments other than depression. Besides its use in treating depression, (*R*)-ketamine also holds significant potential in the prevention and treatment of perioperative anesthesia, ischemic stroke, cognitive disorders, Parkinson’s disease, multiple sclerosis, osteoporosis, substance use disorders, inflammatory diseases, COVID-19, and organophosphate poisoning.

In addition to its unique role in the treatment of depression, (*R*)-ketamine has been shown to have preventive, therapeutic and developmental potential in cognitive disorders, perioperative anaesthesia, cerebral ischaemic stroke, Parkinson’s disease, multiple sclerosis, osteoporosis, substance use disorders, inflammatory disorders, COVID-19, organophosphorus poisoning, etc. (Figure 2), and the related mechanism of action may be more similar to the antidepressant mechanism of (*R*)-ketamine. The mechanism of action may be more similar to the antidepressant mechanism of (*R*)-ketamine, while the mechanism other than the antidepressant effect needs to be explored by further studies due to the limitation of fewer studies at present. In addition, with further research, we believe that (*R*)-ketamine will have more new indications and application potentials to be explored by researchers around the world in the future.

Finally, despite the superior safety profile and fewer side effects of (*R*)-ketamine compared to (*S*)- and (*R*, *S*)-ketamine, it is essential to focus on potential negative issues arising from prolonged use or excessive dosage. For example, it remains to be investigated whether (*R*)-ketamine may present analogous problems to those associated with (*R*, *S*)-ketamine, such as cognitive impairments induced by neurotoxicity, diminished therapeutic efficacy due to drug tolerance, drug addiction and abuse resulting from psychological dependence, and the potential link between ulcerative cystitis and bladder cancer. These questions need to be systematically addressed in subsequent research studies.
